# Effect of final irrigation protocols on the structural integrity and mechanical properties of the root dentine

**DOI:** 10.1590/1807-3107bor-2024.vol38.0072

**Published:** 2024-12-09

**Authors:** Julia Menezes Savaris, Maria Eduarda Paz Dotto, Lucas da Fonseca Roberti Garcia, Emmanuel João Nogueira Leal da Silva, Bruno Alexandre Pacheco de Castro Henriques, Cleonice da Silveira Teixeira, Eduardo Antunes Bortoluzzi

**Affiliations:** a Universidade Federal de Santa Catarina – UFSC, Postgraduate Program in Dentistry, Florianópolis, SC, Brazil.; b Universidade Estadual do Rio de Janeiro – UERJ, School of Dentistry, Department of Endodontics, Rio de Janeiro, RJ, Brazil.; c University of Louisville, Division of Endodontics, Department of Diagnosis & Oral Health, Louisville, KY, USA.

**Keywords:** Endodontics, Ultrasonics, Root Canal Irrigants, Dentin

## Abstract

This study compared the effects of different final irrigation protocols on the mechanical properties and structural integrity of root dentine. One-hundred eight teeth were instrumented and irrigated with sodium hypochlorite (NaOCl) using conventional irrigation (CI). Teeth were distributed into four groups based on final irrigation protocols: Control Group (CG): 17%EDTA/CI + H_2_O; G1: 2.5%NaOCl/Passive Ultrasonic Irrigation (PUI) + EDTA/PUI + NaOCl/PUI (60s each); G2: EDTA/PUI + NaOCl/PUI (30s each); G3: EDTA/PUI + NaOCl/PUI + H_2_O/PUI + CHX/PUI (30s each). Four tests were conducted: three-point flexural strength test, Scanning Electron Microscopy analysis, microhardness assessment, and the push-out bond strength (POBS) of the filling material to the root dentine. Data were analyzed using ANOVA and Tukey tests (Flexural strength), and Student t-test (Microhardness). Erosion scores and POBS were analyzed by Kruskal-Wallis and Dunn tests (α = 0.05). Results indicated no significant differences in flexural strength (p > 0.05) among groups. CG exhibited the lowest erosion scores in the cervical third. In the middle third, CG had lower scores than G1 and G2, while in the apical third, CG had lower scores than G1 (p < 0.05). Microhardness values decreased following the protocols (p < 0.05), except for the CG (p > 0.05). G2 displayed higher POBS values in the middle and apical thirds (p < 0.05). The protocols did not significantly influence the flexural strength of root dentine. However, they did affect microhardness and promoted greater erosion. The best results for POBS were observed when the final irrigation involved the sequential use of EDTA and NaOCl employing PUI for 30 seconds in each solution.

## Introduction

Irrigation procedures are essential to achieve proper cleaning and disinfection, especially in complex anatomical areas that cannot be mechanically touched during root canal preparation procedures.^1^ Sodium hypochlorite (NaOCl) is the most used endodontic irrigant, mainly due to its excellent antimicrobial and tissue-dissolving activity.^2,3^ While the organic portion of the smear layer can be removed by NaOCl^4^ chelating agents, such as ethylenediaminetetracetic acid (EDTA) are indicated to remove its inorganic portion.^5^ Chlorhexidine gluconate (CHX) has also been recommended as a root canal irrigant,^6^ because of its broad-spectrum antimicrobial action^2^ and substantivity property, which provides prolonged antimicrobial action.^6^ However, due to the lack of tissue-solving ability^3^ it has been suggested mainly as a final irrigant solution to be used after NaOCl irrigation protocols.^7^

Activation methods associated with irrigant solutions are also used to improve the cleaning and disinfection outcomes.^8^ Among the different activation methods, passive ultrasonic irrigation (PUI) is one of the most widely laboratorial and clinically tested.^8-10^ The cavitation and acoustic streaming generated by the ultrasound provide more effective irrigation of lateral canals and isthmus^8^ and favor the smear layer removal.^10^

Although PUI is currently accessible and popular, its mode of application has not yet been well defined in a standardized final irrigation protocol. Analyzing the pertinent literature, the different use of PUI can be highlighted in two final irrigation protocols^10-16^. In one of them, the authors have recommended a sequence of activation of NaOCl, EDTA and NaOCl in 3 cycles of 20 seconds each.^11,13,14^ In others, it was proposed to reduce the activation time, *i.e.*, only one cycle of 30 seconds, in order to minimize possible harmful effects on the root dentine, without impairing the benefits of PUI.^10,15,16^

Despite the proven benefits, these protocols may increase the potential of the endodontic chemical substances in inducing changes in the structure and composition of the root dentine.^17^ These modifications might affect dentine microhardness^18^ and flexural strength,^19^ with a possible negative impact on the tooth fracture resistance.^20^ In addition, these protocols may interfere with the interaction between the filling material and the intraradicular dentine, hindering proper sealing.^21^ However, there is no consensus in the literature concerning the most appropriate irrigation protocol to be used.^22^ This justifies further in-depth studies comparing different irrigation protocols, varying the sequence of solutions used, and the duration of PUI.

Therefore, the objective of this study was to compare two protocols using the EDTA and NaOCl sequence with different PUI durations, and a third protocol that uses, in addition to EDTA and NaOCl, chlorhexidine as the final irrigating solution, with regard to the structural integrity and mechanical properties of root dentin. The null hypotheses tested were that there would be no significant difference among the different final irrigation protocols regarding: the changes in the root dentine structure; the flexural strength; the microhardness; and on the push-out bond strength (POBS) of the filling material to the intraradicular dentine.

## Methods

This research was approved by the local Human Research Ethics Committee (CAAE: 14842919.0.0000.0121).

### Sample size calculation and selection

The sample size was estimated with the G*Power 3.1.9.4 software (Heinrich-Heine Universität, Düsseldorf, Germany), based on data from previous studies^15,23,24^, with statistical power of 80%, alpha level of 5%, and effect size of 1.8. To observe significant differences, each group should contain at least 12 specimens for the flexural strength test and erosion analysis, 3 specimens for the microhardness test, and 12 specimens for the POBS test.

Therefore, 108 human teeth with a single and straight canal, and fully formed roots were selected, confirmed with digital radiographs. Teeth with calcifications, cracks, curvatures, with an apical diameter larger than a size 20 file, or previous root canal treatment were excluded from the final sample. Of these, 48 mandibular premolars were used for erosion evaluation and flexural strength test, 12 mandibular incisors for microhardness test and 48 mandibular premolars for POBS test. After cleaning with ultrasonic scalers and rinsed with distilled water, the teeth were stored in 0.05% thymol for a week to prevent bacterial growth.

### Scanning Electron Microscopy (SEM) analysis

**Figure 1:**
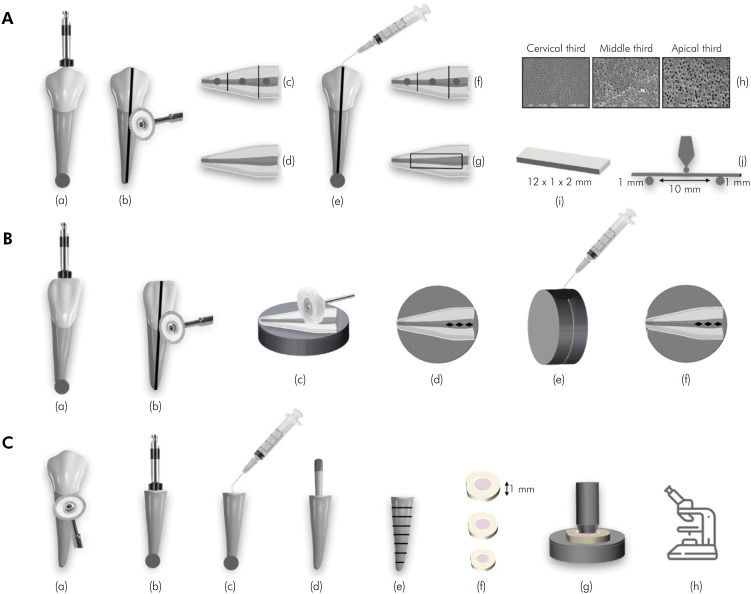
Schematic illustration of mechanical tests. (A) SEM analysis and three-point flexural strength test: root canal preparation (a); longitudinal grooves (b); one tooth half with marks in the root canal lumen for low-vacuum SEM (c); another tooth half for posterior analysis (d); tooth halves reassembled and application of final irrigation protocols, in 4 groups (e); high-vacuum SEM evaluation (f); cut of root dentine bean (g); SEM images for dentine surface evaluation of all thirds (h); dentine beans with previously defined dimensions (i); three-point flexural strength test (j). (B) Microhardness test: root canal preparation (a); longitudinal grooves (b); inclusion and polishing of each tooth half (c); indentations on root canal lumen – initial evaluation (d); reassemble of tooth halves and applications of the final irrigation protocols, in 4 groups (e); new microhardness measurement (f). (C) Push-out bond strength test: crowns removal (a); root canal preparation (b); final irrigation protocols, in 4 groups (c); obturation procedures (d); dentine slices cutting (e); selection of one slice per third (f); application of the force until the displacement of the filling material (g); classification of the failure modes (h).

Forty-eight mandibular human premolars were used to evaluate changes on the root dentine structure (Figure 1A). After coronal access, the tooth length was obtained by inserting a size 10 K-file (Dentsply-Malillefer, Ballaigues, Switzerland), until the tip was visualized in the apical foramen. The working length (WL) was obtained by subtracting 1 mm from the tooth length.

The apical portion of each tooth was sealed with sticky wax (New Wax; TechNew, Rio de Janeiro, Brazil) to avoid extravasation of the irrigating solutions. Root canal preparation was performed using Reciproc® R40 instruments (VDW, Munich, Germany) (Figure 1A-a). During preparation (10 minutes), each canal was irrigated with 6 mL of 2.5% NaOCl (Asfer, São Paulo, Brazil) using a single sided port NaviTip 30-gauge tip (Ultradent Products Inc., South Jordan, USA) coupled to a 5-mL syringe. The NaviTip was calibrated 2mm short of the WL. Irrigant delivery was accomplished using back-and-forth movements of 2–3 mm amplitude. Apical patency was maintained by inserting a size 10 K-file in the WL. On completion of instrumentation, each canal was dried by aspiration using a suction cannula.

Then, a master Reciproc^®^ R40 gutta-percha cone (VDW) was inserted into the canal without using sealer. Longitudinal grooves were made on the buccal and lingual external surfaces of each tooth using a 22-mm diameter diamond double-sided disk (KG Sorensen, São Paulo, Brazil) (Figure 1A-b). The disk was used at low speed until a faint hue of pink gutta-percha was seen, to avoid accidental contamination of the canal space by cutting debris. The grooves were cleaned with air-water spray.

Each tooth was split gently along the grooves with a hammer and a chisel. One of the 2 tooth-halves was selected for SEM. Each tooth-half was virtually divided into thirds (cervical, middle and apical) by making 3 marks external to the root canal and perpendicular to its longitudinal axis with a fine-tip pen (Figure 1A-c). The marks also served as a reference for the preparation of three circular indentations, located between the thirds of the root canal space. Each circular indentation was prepared with a 1-mm diameter dermatological micro punch (Biopsy Disposable Dermal Punch, Miltex, York, USA).^16^ A jet of air and water was applied over the indentation marks to remove cutting debris. The specimens were kept in a desiccator containing anhydrous silica for 48h to remove moisture.

Without any coating or additional preparation, the specimens were examined with SEM operated using the low-vacuum mode (TM3030; Tabletop Microscope, Hitachi, Tokyo, Japan) (Figure 1A-c). To obtain images of the demarcated circumference, a 100x image was used to view each circular indentation in full. Without changing the position of the specimens, two images were obtained at 500x and 1000x magnifications. Six images were obtained for each specimen prior to irrigation (i.e., 2 images for each canal-third). These initial images show the condition of the root canal walls before the final irrigation protocols and were used to compare to the final images, after the protocols.

The halves of each tooth were re-assembled. The grooves previously created for cleavage were filled with light curing resin (Topdam; FGM, Joinville, Brazil) to stabilize the tooth-halves, according to previously described and tested^15^. The apical region was again sealed with sticky wax (Figure 1A-e).

**Figure 2:**
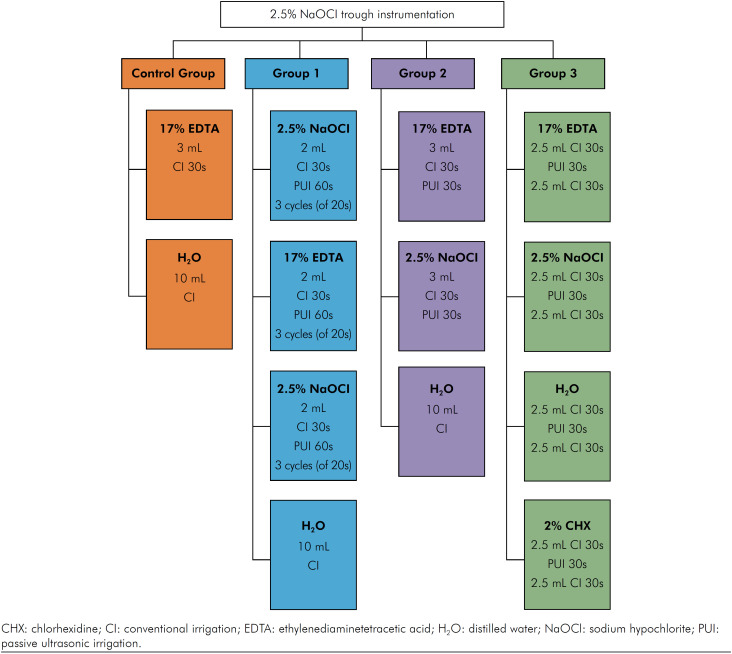
Final irrigation protocols according to the different groups showing the sequence, method, volume and duration of each solution.

CHX: chlorhexidine; CI: conventional irrigation; EDTA: ethylenediaminetetracetic acid; H_2_O: distilled water; NaOCl: sodium hypochlorite; PUI: passive ultrasonic irrigation.

The teeth were randomly distributed into 4 groups (n = 12) according to the final irrigation protocols. The sequence, method, volume and duration of the solutions used in each final irrigation protocol are explained and detailed in Figure 2.

Conventional irrigation (CI) was performed using a NaviTip 30-gauge tip (Ultradent) coupled to a 5-mL syringe. The NaviTip (Ultradent) was calibrated 2mm short of the WL. Irrigant delivery was accomplished using back-and-forth movements of 2-3 mm amplitude. PUI was performed using an Irrisonic E1 tip, without cutting power (Helse, São Paulo, Brazil) positioned 1 mm below the WL, activated by the ART-P6 Compact Piezoelectric Scaler ultrasonic unit (Bonart Medical, New Taipei City, Taiwan) at a 20% power, avoiding contact with the root canal walls.

**Figure 3:**
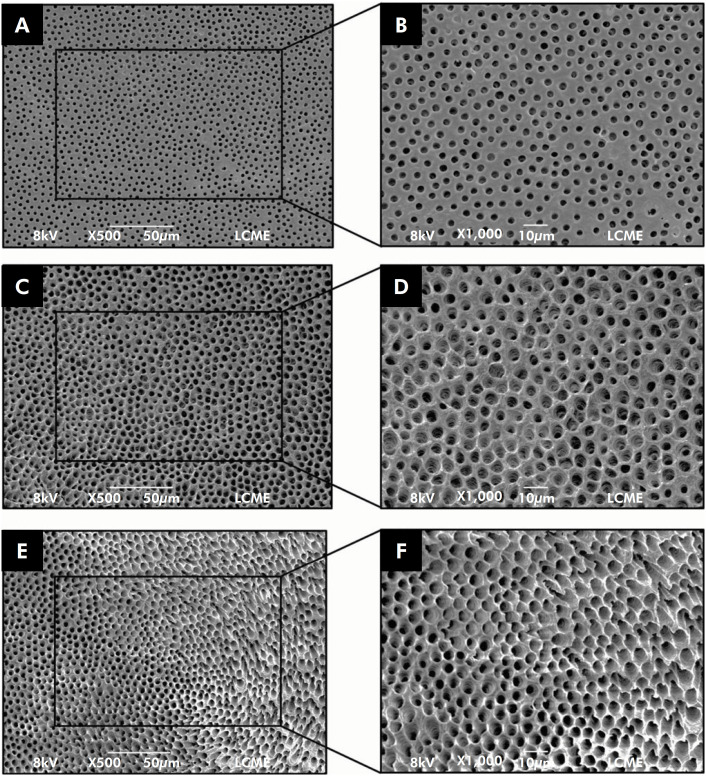
Representative SEM images of the erosion scores: (a) score 1 in a 500x; (b) score 1 in a 1000x; (c) score 2 in a 500x; (d) score 2 in a 1000x; (e) score 3 in a 500x; (f) score 3 in a 1000x.

After final irrigation protocols, the teeth were separated again. The same half previously analyzed, was placed in a silica desiccator for 48h and sputtered with gold to a high-vacuum SEM observation (Figure 1A-f). Two images per third were taken in the same places previously marked (Figure 1A-h). For the dentine surface evaluation, regarding the occurrence of erosion, the images acquired were scored by two previously calibrated and blinded examiners, according to the Torabinejad et al.^25^ methodology: a) without erosion (all dentinal tubules with normal size and appearance); b) moderate erosion (dentinal tubules of the peritubular dentine with erosion); c) severe erosion (intertubular dentine is eroded and the dentinal tubules communicate with each other) (Figure 3). The examiners’ calibration took place at two different times, one week apart. Twelve random SEM images were selected and they assigned the scores mentioned above. The intra-examiner and inter-examiner agreement was calculated with Kappa test at the two different time-periods.

### Three-point flexural strength test

The half not used in the dentine erosion evaluation was prepared and submitted to the three-point flexural strength test (Figures 1A-d and g). A root dentine slice with 12mm x 2mm x 1mm was obtained from each specimen, using a cutting machine (Isomet 1000; Buehler, Illinois, USA) with a diamond saw (Buehler) at low speed and under copious water cooling (Figure 1A-i). The measurements were confirmed with a digital caliper (16 ES; Carl Mahr Esseingen GmbH, Göttingen, Germany). The slices were kept in distilled water until the test was performed.

Each dentine slice was subjected to a three-point bending test (Figure 1A-j). using a Universal Testing Machine (Instron Model 4444; Norwood, USA), as previously described^26,27^. The maximum force required to fracture each specimen was noted. The flexural strength was calculated in Mpa, according to the formula: $\text{Flexural strength (MPa)}=\text{(3.P.L)/(2.b}{\text{.d}}^{\text{2}}\text{)}$ , where P = load to fracture (N), L = support length (mm), b = beam width (mm), and d = beam thickness (mm).

### Microhardness test

For this experiment, 12 mandibular incisors were selected, following the previously described inclusion criteria. The access, chemo-mechanical preparation and longitudinal cuts were performed as previously described (Figure 1B-a and b).

The halves of each tooth were included in epoxy resin discs. After the resin setting, each root canal side was sequentially polished with silicon carbide sandpapers (3M/ESPE, Saint Paul, Minnesota, USA) with progressively increasing grit sizes (#400, 600, 800 and 1200), under constant irrigation (Figure 1B-c). This initial procedure was carried out taking care to preserve a sufficient root canal space, so that the final irrigation protocols could be performed. To ensure that, the central portion of the cervical third was glossy and flattened, a final polishing procedure was performed with alumina-based pastes in decreasing order of granulation (1.0, 0.5 and 0.03 μm) (Buehler). At each change of abrasive paper or paste, the specimens were extensively rinsed in running water. Surface visualization was done under an operating microscope at 24x. They were dried with air jets and stored with 95% humidity at 37°C until the test was carried out.

Dentine microhardness was measured with a Knoop indenter at x40 magnification (Shimadzu, Kyoto, Japan) under a 100g load and a 5s dwell time. For the initial evaluation, three indentations were made parallel on the root canal lumen on the cervical third (Figure 1B-d). The first indentation was made 1000µm from the root canal entrance, and two other indentations were made at 200µm from each other.^27^ The average length of the two diagonals was used to calculate the microhardness value (Knoop number hardness [KHN]). The hardness value for each specimen was obtained as the average of the results for the three indentations.

Then, the halves of each tooth were reassembled and stabilized with stick wax. The final irrigation protocols were applied (Figure 1B-e). Teeth were separated again, and a new microhardness measurement was performed, as previously described (Figure 1B-f).

### Push-out bond strength test

Forty-eight mandibular human premolars were used in this test. The crowns were removed with a 7020 double-sided diamond disc 22 mm in diameter and 0.1 mm thick (KG Sorensen) and roots measuring approximately 16 mm were obtained (Figure 1C-a). The chemo-mechanical preparation and the final irrigation protocols were performed as previously described (Figure 1C-b and c).

After the final irrigation, the root canals were dried with absorbent paper cones (VDW). Each root canal was filled using a Reciproc^®^ R40 gutta-percha master cone (VDW) and AH Plus sealer (Dentsply-Maillefer) (Figure 1C-d). In addition, FF accessory cones (Dentsply-Maillefer) were added in the middle and cervical thirds by lateral compaction, with the aid of an endodontic finger spreader (Dentsply-Maillefer). Digital radiographs were taken to confirm the quality of the filling (New Ida; Dabi Atlante, São Paulo, Brazil). The material excess was removed, and the cervical access was sealed with temporary restorative material (Coltosol; Còltene, Altstätten, Switzerland). All specimens were stored at 37°C for 7 days.

Then, roots were included in self-curing acrylic resin sticks (Jet Classic; São Paulo, Brazil), to be cross-sectioned in perpendicular cuts along the axis of the canal (Figure 1C-e). The 1±0.01mm thick dentine slices were obtained with a 152.4 mm x 0.5 mm x 12.7 mm diamond cutting disc (Buehler), coupled to an Isomet 1000 high precision metallographic cutter (Buehler), with a weight of 150 g and speed of 250 rpm, under constant refrigeration. The thickness of each dentine slice was checked using a Mahr 16 ES digital caliper (Carl Mahr Esseingen GmbH). A slice per third was selected (Figure 1C-f).

The specimens were fixed on a metallic base, coupled to the lower portion of the Universal Testing Machine (Instron). A metal rod, with an active tip selected according to the diameter of the canal, in order to cover the largest possible portion of the filling material (between 0.47 mm to 1.3 mm), was fixed in the upper part of the machine and activated in the apico-cervical direction, with a crosshead speed of 0.5 mm/min, until the displacement of the filling material (Figure 1C-g). The force required for displacement was measured in kilonewtons (kN), transformed into Newtons (N) and divided by the lateral area (SL) of the filling (in mm²) to be converted into MPa. The SL was calculated using the following formula: $\text{SL}=\text{π}(\text{R}+\text{r})\sqrt{{\text{h}}^{2}+{\left(\text{R}-\text{r}\right)}^{2}}$ where, SL = sealer bonding area; π = 3.14; R = radius of the coronal canal (mm); r = radius of the apical canal (mm); h = thickness of the slice (mm).

After the test, the dentine slices were examined under a stereomicroscope (SteREO Discovery. V12, Carl Zeiss, Jena, Germany), with a 40x magnification (Figure 1C-h), to classify the failure modes: a) adhesive failure, with the absence of root filling material on the dentinal walls; b) cohesive failure, with the presence of root filling material on the dentinal walls; and c) mixed failures when adhesive and cohesive failures were observed in the same sample. If there was a doubt about the failure type, the sample was submitted to a SEM evaluation, with an 80x magnification.

### Statistical analysis

The data normality was checked using Shapiro-Wilk test. The normal data (flexural strength and microhardness) were submitted to ANOVA and Tukey's post-hoc test. The microhardness data were submitted to the dependent Student's t test, to compare the values before and after the final irrigation protocols. An ANOVA test was conducted among the four groups to compare the percentage of microhardness reduction after the final irrigation protocols.

Non-normal data (dentine erosion scores and POBS) were evaluated using Kruskal-Wallis and Dunn multiple comparison tests. Statistical analysis was performed using JAMOVI 1.2.27 software (public domain) and BioEstat 5.0 (Mamirau Foundation, Belem, Brazil). The level of significance adopted was 95%.

## Results

### Changes on the root dentine structure (dentine erosion evaluation)

The Kappa test demonstrated outstanding agreement –intra and inter-examiners, with the respective values exceeding 0.85 and 0.90, correspondingly.

The average rank, median of the scores, and the results obtained from the comparison of the canal thirds in the 4 groups are summarized in Table 1. CG (EDTA/CI + H_2_O/CI) had the lowest dentine erosion scores in the cervical third (p < 0.05). The middle third of CG (EDTA/CI + H_2_O/CI) presented lower scores than G1(NaOCl/PUI + EDTA/PUI + NaOCl/PUI) and G2 (EDTA/PUI + NaOCl/PUI) (p < 0.05), while on the apical third a lower dentine erosion score was observed only when CG (EDTA/CI + H_2_O/CI) was compared to G1 (NaOCl/PUI + EDTA/PUI + NaOCl/PUI) (p < 0.05). In the intragroup analysis, none of the groups showed difference (p > 0.05). Table 2 shows the severity of erosion that occurred in the different groups and canal-thirds.

**Table 1:** Dentine erosion mean rank scores and median of scores (in parentheses); mean and standard deviation (±SD) values of flexural strength (MPa) in the three-point test; mean and standard deviation, and percentage of difference in root dentin Koop Harness Numbers (KHN), before and after the final irrigation protocols.**

Groups*	Dentine erosion mean rank scores (median of scores)			Flexural Strength (Mean MPa ± SD)	Root Dentine Microhardness (Mean KHN ± SD)		
	Cervical	Middle	Apical		Before	After	% of Reduction
CG: EDTA/CI + H_2_O/CI	8.58 (1.0)^Aa^	9.00 (1.0)^Aa^	13.33 (1.0)^Aa^	233.50 ± 36.91ª	45.91 ± 13.41^Aa^	38.55 ± 8.41^Aa^	14.24^a^
G1: NaOCl/PUI + EDTA/PUI + NaOCl/PUI + H_2_O/CI	36.08 (3.0)^Ab^	32.16 (3.0)^Ab^	34.70 (3.0)^Ab^	198.93 ± 36.74ª	39.54 ± 7.51^Aa^	33.51 ± 5.89^Ba^	15.01^ab^
G2: EDTA/PUI + NaOCl/PUI + H_2_O/CI	29.00 (2.5)^Ab^	34.62 (3.0)^Ab^	24.16 (1.5)^Aa^	204.31 ± 37.88ª	46.81 ± 10.97^Aa^	36.71 ± 8.64^Ba^	21.32^ab^
G3: EDTA/PUI + NaOCl/PUI + H_2_O/PUI + CHX/PUI	24.33 (2.0)^Ab^	22.20 (2.0)^Aa^	25.79 (2.0)^Aa^	209.56 ± 64.89ª	49.52 ± 11.84^Aa^	33.35 ± 14.45^Ba^	33.54^b^

CG: control group; CHX: chlorhexidine; CI: conventional irrigation; EDTA: ethylenediaminetetracetic acid; H_2_O: distilled water; NaOCl: sodium hypochlorite; PUI: Passive Ultrasonic Irrigation; SD: standard deviation.

* CG: CI each solution; G1: CI + PUI (60s) each (3 cycles of 20s); G2: CI + PUI (30s) each; G3: CI + PUI (30s) + CI, each solution. For more details of the final irrigation process, see Figure 2.

** Equal uppercase superscript letters in the rows indicate that there is no difference in the same group for each variable (p > 0.05). Equal lowercase superscript letters in the column indicate that there is no difference among the groups for each analyzed variable (p > 0.05).

**Table 2:** Distribution of erosion levels within the experimental irrigation groups* and root canal thirds.

Erosion		CG		G1		G2		G3		Total (n)
		EDTA/CI + H_2_O/CI		NaOCl/PUI + EDTA/PUI + NaOCl/PUI + H2O/CI		EDTA/PUI + NaOCl/PUI + H_2_O/CI		EDTA/PUI + NaOCl/PUI + H_2_O/PUI + CHX/PUI		
		n	%	n	%	n	%	n	%	
Cervical third										
	No erosion	11	91.6	0	0	0	0	3	25.0	14
	Moderate	1	8.3	1	8.3	6	50.0	4	33.3	12
	Severe	0	0	11	91.6	6	50.0	5	41.6	22
Middle third										
	No erosion	12	100	1	8.3	0	0	4	33.3	17
	Moderate	0	0	3	25.0	3	25.0	5	41.6	11
	Severe	0	0	8	66.6	9	75.0	3	25.0	20
Apical third										
	No erosion	11	91.6	2	16.6	6	50.0	4	33.3	23
	Moderate	1	8.3	1	8.3	2	16.6	5	41.6	9
	Severe	0	0	9	75.0	4	33.3	3	25.0	16

CG: control group; CHX: chlorhexidine; CI: conventional irrigation; EDTA: ethylenediaminetetracetic acid; H_2_O: distilled water; NaOCl: sodium hypochlorite; PUI: Passive Ultrasonic Irrigation; SD: standard deviation.

* CG: CI each solution; G1: CI + PUI (60s) each (3 cycles of 20s); G2: CI + PUI (30s) each; G3: CI + PUI (30s) + CI, each solution. For more details of the final irrigation process, see Figure 2.

### Three-point flexural strength test

Table 1 shows the flexural strength (Mpa) of the rectangular dentine beams submitted to the three-point bending test. There was no statistical difference among groups (p > 0.05).

### Microhardness test

Table 1 contains the mean and standard deviation (±) values of root dentine microhardness, before and after the different final irrigation protocols. No differences in microhardness were observed among the groups, before and after final irrigation protocols (p > 0.05). Final irrigation protocols reduced root dentine microhardness in all experimental groups (p < 0.05); however, no differences were observed in the CG (EDTA/CI + H_2_O) before and after final irrigation (p > 0.05). Also, the percentage of reduction of the microhardness values is presented in Table 1. The CG (EDTA/CI + H_2_O/CI) presented the lowest percentage of reduction (14.24%) and it had statistically difference from G3 (EDTA/PUI + NaOCl/PUI + H_2_O/PUI + CHX/PUI) (p = 0.044). No difference was observed among the other groups (p > 0.05).

### Push-out bond strength test

The push-out bond strength means, and standard deviation values are shown in Table 3. G2 (EDTA/PUI + NaOCl/PUI) showed higher POBS values in the middle and apical thirds (p < 0.05), while no differences were observed in the cervical third (p > 0.05). The failure modes observed in each group were also described in Table 3. Except for G3 (EDTA/PUI + NaOCl/PUI + H_2_O/PUI + CHX/PUI), which demonstrated a higher percentage of mixed failures, cohesive type failures were the most predominant in the other tested groups.

**Table 3:** Mean values and standard deviation of the push-out bond strength (Mpa) for the different final irrigation protocols in relation to the root thirds and groups; percentage (%) of failure types present in different groups.

Groups*	Thirds (MPa)**			Failure types (%)		
	Cervical	Middle	Apical	Adhesive	Cohesive	Mixed
CG: EDTA/CI + H_2_O/CI	3.06 ± 1.92^Aa^	3.01 ± 1.59^Aa^	3.06 ± 1.38^Aa^	0	61.76	38.23
G1: NaOCl/PUI + EDTA/PUI + NaOCl/PUI + H_2_0/CI	3.16 ± 1.11^Aa^	4.92 ± 3.41^Aa^	4.37 ± 1.94^Aa^	0	72.22	27.77
G2: EDTA/PUI + NaOCl/PUI + H_2_O/CI	5.50 ± 3.73^Aa^	6.54 ± 2.85^Ab^	7.49 ± 3.61^Ab^	8.33	52.77	38.88
G3: EDTA/PUI + NaOCl/PUI + H_2_O/PUI + CHX/PUI	3.92 ± 1.49^Aa^	3.91 ± 2.47^Aa^	3.94 ± 1.93^Aa^	2.77	33.33	63.88

CG: control group; CHX: chlorhexidine; CI: conventional irrigation; EDTA: ethylenediaminetetracetic acid; H_2_O: distilled water; NaOCl: sodium hypochlorite; PUI: Passive Ultrasonic Irrigation; SD: standard deviation.

* CG: CI each solution; G1: CI + PUI (60s) each (3 cycles of 20s); G2: CI + PUI (30s) each; G3: CI + PUI (30s) + CI, each solution. For more details of the final irrigation process, see Figure 2.

** Equal lowercase letters in the columns indicate that there is no difference among groups (p > 0.05). Equal uppercase letters in the rows indicate that there is no difference among the thirds of the group (p > 0.05).

## Discussion

The present study evaluated the effect of three different final irrigation protocols on the structural integrity and mechanical properties of root dentine. While no differences were observed for flexural strength and microhardness, differences were observed for dentine erosion and POBS. Therefore, the null hypothesis was partially rejected.

This study was carried out due to the criticism found in the literature about the possible deleterious consequences of the use of EDTA associated with NaOCl in the dental structure.^27^ Two final irrigation protocols were chosen^10-16^ and a third was experimental using CHX. The differences between the first two were the PUI agitation time and the order in which the solutions were used. Both protocols may cause dentine erosion.^14,16^ Theoretically, when root dentine erosion occurred, adverse effects on dentine mechanical properties can be identified.^27^ The mineral content degradation reduces the microhardness and flexural resistance of dentine, which, in turn, predisposes the root to vertical fractures after root canal treatment.^17,20^ In the present study, the irrigation protocols used in all experimental groups caused higher levels of erosion when compared to the CG, which used only EDTA for final irrigation, which agrees with previous studies.^17,19^ The time spent in the EDTA irrigation, in our study, for the CG was chosen based on the results of a previous one.^10^ Despite some slight differences observed between the experimental groups, in general, they behaved very similarly.

The three-point flexural strength test may be related to dentine's certain mechanical properties, such as the tooth's resistance to fracture, its flexibility, and degree of elasticity.^27^ If altered, it may reflect a more fragile structure and predispose the tooth to vertical fracture.^19^ Exposing the dentine surface to NaOCl and EDTA causes a degradation, which might explain the change in flexural strength when irrigation protocols were used.^28^ The combined removal of the inorganic and organic content gives rise to damaging effects on the peritubular and intertubular dentine.^29^ However, although the protocols of the present study used sequential irrigation of EDTA and NaOCl, the results showed no statistical difference among the control and the three experimental groups. The sequential use of EDTA and NaOCl was probably too short to disclose an impact on the mechanical dentine properties as also detected by previous studies.^19,30^ When a brief exposure time was used (10 minutes for NaOCl and 2 minutes for EDTA), it did not negatively impact in flexural strength. In accordance, Zhang et al.^19^ observed that flexural strength only reduced after the use of 5.25% NaOCl for more than 60 minutes. However, when groups were irrigated with 5.25% NaOCl for a time equal to 60 minutes^31^ or 120 minutes, followed by irrigation with EDTA,^19^ a significant reduction in flexural strength was detected. Another study also detected that the use of PUI associated with an increased irrigation time (30 to 60 minutes) might have a negative effect on flexural strength even with low NaOCl concentrations.^32^ The methodological differences in these studies make it difficult to make further comparisons of our results, such as: dentine used in these tests belonged to the tooth crown,^19,31^ and it has a different structural configuration than root dentine, and immersion of specimens in the solutions were used.^19,31,32^

The microhardness was longitudinally analyzed to assess the difference between values before and after the use of the different final irrigation protocols.^33^ In addition, microhardness was measured in the root canal lumen. During irrigation, the solutions first come into contact with the superficial dentine of the root canal lumen and then diffuse through the dentinal tubules^18^. Knoop method was used, because it has greater sensitivity to textures and small defects on surfaces, being used to evaluate dentine substrate.^18,34^ There was no difference between the initial microhardness values among groups. However, all groups, except the CG (EDTA/CI + H_2_O/CI), showed a statistically significant difference between the initial and the final microhardness values. Moreover, the CG presented the lowest percentage of reduction among the groups and it was statistically different from G3. This finding corroborates the results of other studies, which showed that the sequential use of EDTA and NaOCl solution may alter the dentine microhardness.^17,18,34^ Changes in the dentinal microhardness seem to be associated with the occurrence of erosion.^34^

The push-out bond strength results showed that the G2 (EDTA/PUI + NaOCl/PUI) had higher bond strength values at the middle and apical thirds when compared with other tested groups. As noted in previous studies, the irrigation protocol used in G1 (NaOCl/PUI + EDTA/PUI + NaOCl/PUI) and G2 (EDTA/PUI + NaOCl/PUI) effectively removed the smear layer.^10,12,15^ The removal of this layer has a positive impact on the penetration of sealers within the dentinal tubules.^21,24^ A proper cleaning increases the exposed and patent dentinal tubules, which provides more covalent bonds between the sealer and the amino groups of the dentine collagen matrix^35^ and consequently, results in greater adhesion strength. On the other hand, G1 (NaOCl/PUI + EDTA/PUI + NaOCl/PUI) may not showed interesting results regarding POBS because a prolonged exposure of dentine to NaOCl may negatively affect the bond strength, by disorganizing and irreversibly removing proteins from the root dentine.^36^ Regarding G3 (EDTA/PUI + NaOCl/PUI + H_2_O/PUI + CHX/PUI), even if H_2_O intermediate irrigation was performed, there is still a possibility that the precipitate, formed by the interaction between NaOCl and CHX, was deposited on the root dentine surface. This precipitate behave e is known as a chemical smear layer, and may obstruct the dentinal tubules^37^ and negatively interfere with the sealing ability of the filling material.^38^ In agreement with our study, Topçuoglu et al.^39^ showed that the PUI group showed the highest bond strength values at the cervical and middle thirds. Similar results were also obtained by Ackay et al.^40^. The authors concluded that the activation of the irrigating solution has a positive impact on the filling material adhesion to root dentine.

The failure mode analysis showed a higher incidence of cohesive failures in CG (EDTA/CI + H_2_O), G1 (NaOCl/PUI + EDTA/PUI + NaOCl/PUI), and G2 (EDTA/PUI + NaOCl/PUI). The AH Plus root canal sealer used in the present study has high bond strength to dentine and penetration into the dentinal tubules, which leads to cohesive failure patterns.^40^ The difference in failure pattern observed in G3 (EDTA/PUI + NaOCl/PUI + H_2_O/PUI + CHX/PUI) – the highest percentage of mixed failures - may be attributed to the orange-brown precipitate deposition on the dentine surface. This residual precipitate may have prevented the correct interaction between the filling material and the root dentine.^38^

All tested and experimental protocols caused changes in the root dentine structure, as seen in the decrease of microhardness and occurrence of erosion. Nevertheless, the flexural strength was not compromised. Even so, regarding the tooth resistance to fracture in the face of masticatory efforts, further studies are needed to correlate the final irrigation protocol with this condition. As a laboratory-based study, extracted teeth might not fully represent the variety of conditions found in actual patient populations, affecting the generalizability of the findings. Also, it is challenging to replicate all the complex external factors present during clinical treatment, such as variations in operator technique, environmental conditions, and patient-specific factors. While assessing the mechanical properties of dentine is important, it is also crucial to consider that clinical outcomes depend on a combination of factors, including microbial control and biological responses.^1^ The effectiveness of irrigation depends on the operator training and the dental group, which could introduce variations in the quality of treatment. It is worth mentioning that the use of a dentine beam instead of the entire tooth to assess the flexural strength test could have contributed to the similarity of the obtained results, suggesting that future investigations considering the complete tooth may provide a more comprehensive and accurate insight into the studied subject.

## Conclusions

Within the limitations of this study, it is possible to conclude that the different final irrigation protocols evaluated caused a certain dentine erosion degree and altered its microhardness. However, they did not influence the dentin flexural strength. The final irrigation with EDTA followed by NaOCl, using PUI for 30s in each solution, showed the best bond strength of the filling material to the root canal dentine.
